# Neural Coding of Fundamental Frequency and Processing of Discrete Pitch Accents in Middle Age

**DOI:** 10.1111/ejn.70285

**Published:** 2025-10-30

**Authors:** Jacie R. McHaney, Zhe‐chen Guo, G. Nike Gnanateja, Aravindakshan Parthasarathy, Bharath Chandrasekaran

**Affiliations:** ^1^ Northwestern University Evanston Illinois USA; ^2^ University of Wisconsin‐Madison Madison Wisconsin USA; ^3^ University of Pittsburgh Pittsburgh Pennsylvania USA

**Keywords:** fundamental frequency, middle‐age, pitch accents, prosody, speech perception

## Abstract

Pitch is a prosodic element of speech that speakers dynamically manipulate to convey intention and meaning, making it a powerful cue for spoken language. Neural encoding of fundamental frequency, the primary acoustic cue for pitch, has been shown to deteriorate with age and can occur as early as midlife without overt hearing loss. Here, we systematically examined neural coding of fundamental frequency in middle‐aged and young adult listeners using syllabic and naturalistic speech stimuli. We then examined the extent to which neural processing of pitch accents, phonological units of prosody that convey differences in word prominence in naturalistic speech, differed based on age group. Our findings revealed that middle‐aged adults exhibited reduced neural coding of fundamental frequency to syllabic and naturalistic stimuli compared with younger adults. Middle‐aged adults also demonstrated less distinct neural processing of pitch accents in naturalistic speech, as reflected by greater classification uncertainty in a neural network model classifying pitch accent categories. This finding suggests less specialized cortical processing of suprasegmental prosodic features in the auditory cortex in middle age. Reduced neural coding of fundamental frequency was associated with greater classification uncertainty for pitch accents, linking early auditory deficits to higher order prosodic processing challenges.

## Graphical abstract

Middle‐aged adults exhibited reduced neural encoding of fundamental frequency (F0) in response to both syllabic and naturalistic speech compared with younger adults. They also showed less distinct cortical processing of pitch accents, reflected by increased classification uncertainty in a neural network model. These findings suggest that early‐stage auditory declines in middle‐age may contribute to deficits in suprasegmental prosodic processing.

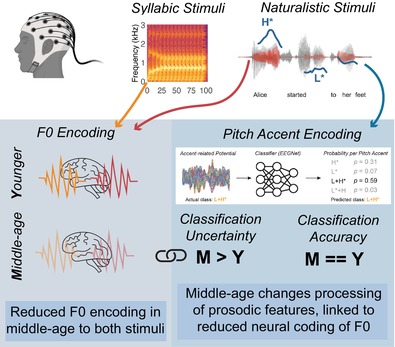

**Abbreviations**
ARP: accent‐related potential
EEG: electroencephalography
F0: fundamental frequency
FFR: frequency‐following response
H: high tonal target
IMF: intrinsic mode function
L: low tonal target
SNR: signal‐to‐noise ratio
SPL: sound pressure level
TRF: temporal response function

## 1 Introduction

Prosody in speech is composed of rhythm, stress, and intonational patterns, which profoundly influence how spoken language is processed and interpreted (Bänziger and Scherer 2005; Gussenhoven et al. 1997; Pell et al. 2009). Prosody is conveyed by multidimensional cues that include pitch, duration, loudness, and timbre. Pitch cues in speech correspond to the frequency of vocal fold vibrations, known as the fundamental frequency (F0). Variations in the F0 can contrast and highlight certain words, shaping their prominence and meaning. In the current study, we used electroencephalography (EEG) to examine neural processing of F0 in syllabic and narrative contexts in middle‐aged and younger adults. We then examined the extent to which neural processing of pitch accents, phonological units of prosody that convey differences in word prominence, differed based on age group. In the following sections, we motivate the key components of the study.

Middle‐aged adults increasingly report speech perception difficulties, despite the absence of hearing loss (Demeester et al. 2012; Helfer and Jesse 2021). To examine the reasons for such speech perception challenges in middle‐age, Guo et al. (2025) used EEG to examine neural representations of speech sounds during listening. Scaffolded by the age‐related neural dedifferentiation model positing that aging leads to less specialized and more correlated activity in the sensory cortex (Koen and Rugg 2019; Park et al. 2004), they hypothesized that middle‐aged adults had reduced specialization of neural responses to distinct segmental speech categories. They found that middle‐aged adults with normal hearing had less robust neural encoding of phonemes that required additional neural sources for processing, suggesting that neural dedifferentiation for speech sounds can occur as early as middle‐age.

In addition to deficits in speech representations, aging is known to be accompanied by a reduction in the representation of voice pitch and pitch related cues (Anderson et al. 2012, 2013; Maruthy et al. 2017; Parbery‐Clark et al. 2012). Using the frequency‐following response (FFR), a scalp recorded potential that reflects phase‐locked neuronal activity in the auditory system (Gardi et al. 1979; Gerken et al. 1975; Worden and Marsh 1968), Anderson et al. (2011) showed that older adults with poor speech perception in noise exhibited reduced neural encoding of F0 when processing speech sounds. While evidence for this relationship in middle‐aged adults is limited, one study reported that middle‐aged adults had poorer neural encoding of F0 compared with younger adults (Ruggles et al. 2012). Moreover, F0 encoding was linked to speech perception in noise performance in younger adults, suggesting that declines in F0 encoding may underlie some of the speech perception challenges observed in middle‐age.

Previous studies using the FFR to examine age‐related declines in neural encoding of F0 used discrete, isolated syllable stimuli (e.g., /da/), which often require hundreds to thousands of repetitions of the stimulus to achieve a robust neural signal. While syllable‐level (e.g., /da/) stimuli can provide informative conclusions on the fidelity of auditory processing, syllable stimuli poorly represent naturalistic listening situations (Hamilton and Huth 2018). In naturalistic speech, F0 changes dynamically to convey intonation (Cole 2015; Ladd 2008) and emotion (Bänziger and Scherer 2005; Pell et al. 2009). The limited ecological validity of static F0 in syllable stimuli compounds the difficulty of inferring speech and prosodic processing challenges from reduced neural coding of F0 in middle age. Recent methodological advances, however, now enable the measurement of neural responses to dynamic F0 in continuous, naturalistic speech (Forte et al. 2017), providing a more realistic framework for studying speech processing. One study using this continuous speech approach found that neural encoding of F0 in naturalistic speech declines in middle‐age (Van Canneyt et al. 2021). However, the extent to which neural coding of F0 in continuous speech reflects the same mechanism captured by isolated syllables is unclear.

**FIGURE 1:**
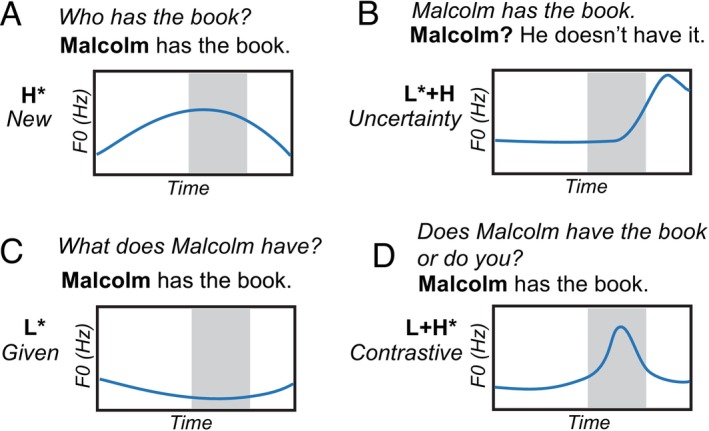
Schematic of F0 contours in four common linguistic pitch accents in English for the word Malcolm. Shaded regions represent the stressed syllable within the accented word. Time is represented along the *x*‐axis, and F0 is represented along the *y*‐axis. (A) The H* pitch accent reflects the identification of salient and new information, with a shallow F0 peak aligned with the stressed syllable. In response to the question, “Who has the book?,” the speaker may emphasize the name “Malcolm” with an H* pitch accent in the sentence and “Malcolm has the book,” denoted by a shallow rise in pitch peaking at the stressed syllable to introduce Malcolm as new information. (B) The L* + H pitch accent reflects uncertainty, represented by a late and steep F0 peak. Malcolm in “Malcolm? He doesn't have the book,” can be spoken with a low to high pitch pattern to express uncertainty. (C) The L* pitch accent captures salient but given information, reflected by a low dipping F0 contour. (D) The L + H* pitch accent indicates contrastive information, with an early and steep F0 peak. Additional pitch accent examples can be found at 10.17605/OSF.IO/9RDFJ.

One advantage of using continuous, narrative speech is the potential to investigate crucial cues that impact spoken language processing, such as linguistic pitch accents. Pitch accents are suprasegmental phonological units of prosody that convey differences in word prominence (Figure 1) through changes in F0 contours (Beckman and Pierrehumbert 1986; Gussenhoven 2004). Typically, speakers use pitch accents sparingly to stress a specific syllable within a word, conveying the speaker's intention, and their selective occurrence makes them crucial in shaping discourse because they do not appear on every word (Pierrehumbert and Hirschberg 1990). Pitch accents are phonologically represented by a high (H*) and low (L*) tonal element aligned with the stressed syllable, defined by the relative pitch and the temporal pitch direction of the F0 contour (Beckman and Pierrehumbert 1986). Pitch accents can also have two tones (H and L) associated with a single prominence‐marking (*) event, known as bitonal pitch accents. In English, the most frequently occurring pitch accent categories are L*, H*, L + H*, and L* + H (Llanos et al. 2021), comprising two single tonal pitch accents and two bitonal pitch accents. In pitch accent notation, the asterisk (*) denotes the tonal target with a specific temporal alignment to the prominent stressed syllable of the word, and the plus (+) in bitonal pitch accents is used to indicate that the two tones are associated for a single unit. Examples of the four pitch accents are described in Figure 1.

Prior work demonstrates that neural processing of the four most common pitch accents can be decoded from EEG responses to continuous speech in younger adults (Llanos et al. 2021). Furthermore, a recent study using stereoelectroencephalography, which measures neural responses collected through electrodes penetrating into the cortex (Abel et al. 2018; Faraji et al. 2020; Parvizi and Kastner 2018), demonstrated that pitch accents are primarily encoded in Heschl's gyrus and are impacted by the neural coding of F0 cues in adolescents and younger adults (Gnanateja et al. 2025). However, relatively little is known about how the neural encoding of pitch accents may change with increasing age. Given that prosodic processing deficits are highly prominent in older adults (Martzoukou et al. 2022), an important question is whether prosodic deficits are present in middle‐aged adults, when risk factors for age‐related hearing loss may still be mitigated.

In the current study, we used EEG to evaluate whether neural coding of F0 is impacted in middle‐aged adults and if it has any impact on downstream neural processing of pitch accents. First, we investigated neural encoding of F0 to both syllables and narrative speech in younger and middle‐aged adults to examine F0 encoding in isolated syllables and in a naturalistic context. Importantly, we sought to replicate and expand upon the findings of Ruggles et al. (2012) of reduced neural encoding of F0 in a larger sample of middle‐aged adults. We predicted middle‐aged adults would demonstrate reduced neural encoding of F0 to both syllabic and continuous speech stimuli, demonstrating that neural coding of F0 is awry in middle age, regardless of stimulus type. To examine the extent to which neural coding of F0 has downstream impacts on neural processing of pitch accents, we trained a convolutional neural network model to classify EEG responses recorded to the narrative speech into pitch accent categories and examined classification accuracy and entropy between the two age groups. We predicted that middle‐aged adults would show poorer neural processing of pitch accents compared with younger adults, providing additional evidence for neural dedifferentiation during speech processing in middle age that was observed for segmental speech features (e.g., phonemes) in Guo et al. (2025). Alternatively, the absence of age‐related differences in neural processing of pitch accents would suggest relative preservation of suprasegmental speech features in middle‐aged adults, as opposed to segmental speech features.

## 2 Material and Methods

### 2.1 Participants

**FIGURE 2:**
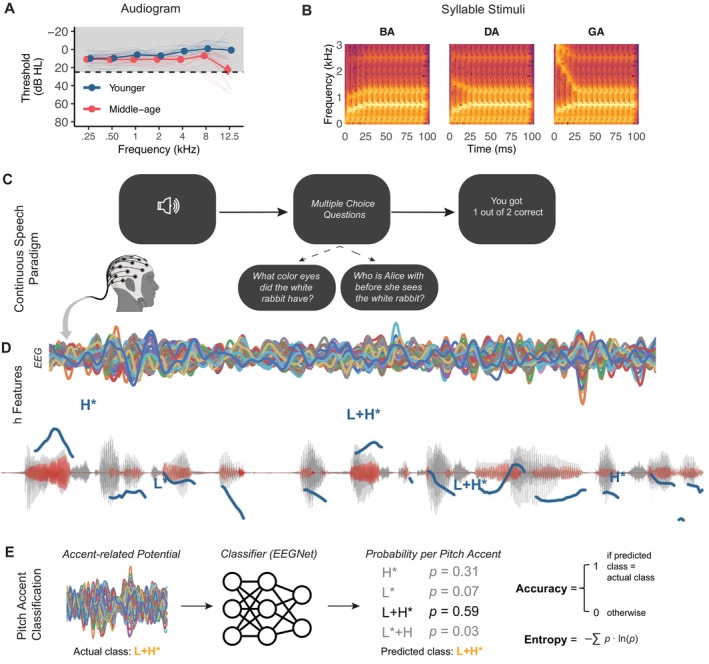
Experimental methods. (A) Average binaural thresholds for all participants split by age group. Individual participant thresholds are denoted by smaller lines. Dashed line represents the threshold cut off used for eligibility screening at 0.25–0.4 kHz. (B) Spectrograms of the first 100 ms for the syllable stimuli used in the FFR portion of the experiment. Each syllable had the same F0 (100 Hz) and primarily differed in the second formant transition. (C) The continuous speech paradigm. EEG was recorded while participants listened to ~1‐min segments of speech. Two multiple‐choice questions were presented after each segment to assess comprehension and attention, with minimal feedback given to indicate how many questions out of the two were answered correctly. (D) We extracted the dynamic F0 waveform (red) from the continuous speech stimuli (grey) to assess neural coding of dynamic F0 the EEG responses (coloured traces). We also extracted time‐locked EEG responses to each instance of the four pitch accents (blue) and calculated an average EEG response for each of the four pitch accents to create an accent‐related potential (ARP). (E) The ARPs at each electrode (coloured traces) were classified using a convolutional neural network (EEGNet) to classify the ARP into one of the four pitch accent categories. The pitch accent category with the highest probability was noted as the predicted class. If the predicted class matched the actual class, the prediction was considered accurate. Model confidence was calculated using Shannon's entropy.

Participants were recruited from the Greater Pittsburgh area for a larger experiment investigating speech perception in noise in aging (Guo et al. 2025; Zink et al. 2025). A subset of participants from this larger study, 20 younger adults (18 females) between the ages of 18–25 years (*M* = 21.550, *SD* = 2.012) and 18 middle‐aged adults (10 females) between the ages of 40–52 years (*M* = 45.389, *SD* = 4.002) completed all required measures reported in this study. All participants were native English speakers with normal cognition as determined by a score ≥ 25 on the Montreal Cognitive Assessment (Nasreddine et al. 2005), and air conduction thresholds ≤ 25 dB hearing level (HL) at 0.25, 0.5, 1, 2, and 4 kHz (Figure 2A). This research study was approved by the Institutional Review Board at the University of Pittsburgh. All participants provided written informed consent.

### 2.2 FFRs to Syllables

FFRs were collected to syllable stimuli to examine neural coding of static F0. The syllable stimuli were synthetically generated /ba/, /da/, and/ga/ syllables (Johnson et al. 2008). The three stimuli were synthesized at a sampling rate of 20 kHz using a Klatt cascade/parallel formant synthesizer (Klatt 1980). Each stimulus was 170 ms long, with a voicing onset at 10 ms. The F0 was identical across stimuli (100 Hz) and consistent throughout. The stimuli had identical formant transition durations (50 ms), a linearly rising first formant (400–720 Hz), and flat fourth (3300 Hz), fifth (3750 Hz), and sixth (4900 Hz) formants. The second (*F*_2_) and third (*F*_3_) formant starting positions differed between stimuli but remained constant after their transition endpoints (*F*_*2*_: 1240 Hz; *F*_*3*_: 2500 Hz). *F*_2_ rose from 900 Hz for /ba/, fell from 1700 Hz for /da/, and fell from 3000 Hz for /ga/. *F*_3_ rose from 2400 Hz for /ba/, fell from 2580 Hz for /da/, and fell from 3100 Hz for/ga/ (Figure 2B).

Participants were seated and watched a muted movie or show of their choice with subtitles during acquisition. The stimuli were binaurally presented in alternating positive and negative polarities via ER3C insert earphones (Etymotic Research, Elk Grove Village, IL) at ~74 dB sound pressure level (SPL). Stimuli were presented with an interstimulus interval of 60 ms. Each syllable stimulus was presented in its own block with 750 repetitions of each polarity (1500 total). The order of stimuli presentation was counterbalanced across participants. Stimuli were presented using the E‐Prime 3.0 software (Schneider et al. 2002).

Electrophysiological responses to each stimulus and listening condition were collected using Ag‐AgCl scalp electrodes, with the active electrode placed at the central zero (Cz) location and reference and ground electrodes at the mastoids. Electrode impedances were ≤ 5 kΩ. Responses were amplified and digitized with the BrainVision actiCHAMP amplifier and EP‐PreAmp using BrainVision PyCorder 1.0.7 (Brain Products, Gilching, Germany). Responses were recorded at a sampling rate of 25 kHz. The electrophysiological data were preprocessed using EEGLAB 14.1.2 (Delorme and Makeig 2004) in MATLAB v2023a (Mathworks Inc., Natick, Massachusetts, United States). Responses were bandpass filtered from 60 to 1500 Hz. Responses were segmented into epochs of 230 ms, which consisted of 20 ms prior to and 210 ms after sound offset. Epochs with response amplitudes that exceeded ±35 μV were rejected.

### 2.3 Continuous Speech EEG Acquisition

EEG was recorded while participants listened to continuous speech (Figure 2C), presented binaurally through ER‐3C insert earphones (Etymotic Research, Elk Grove Village, Illinois) at ~80 dB SPL. Continuous speech stimuli were derived from the public domain audiobook *Alice's Adventures in Wonderland* (Carroll 1865). The story was read in American English by a male speaker and sampled at a frequency of 22.05 kHz. Long speaker pauses were manually truncated to a maximum of 500 ms. The resulting audiobook was divided into segments approximately 60 s in duration (range: 57–65 s) that began and ended with complete sentences. The story was presented in listening conditions of quiet, masked in speech‐shaped noise (SSN) at −2 dB signal‐to‐noise ratio (SNR), and masked in time‐reversed speech at −2 dB SNR. We exclusively focus on results from the quiet condition in the current study to examine the impact of age on neural coding of the dynamic F0 waveform and neural processing of pitch accents without the confounds of noisy environments.

The order of listening conditions was counterbalanced across participants. Fifteen story segments were presented in each condition, and the story segments were always presented in chronological order, regardless of counterbalance order. Two multiple‐choice questions with four answer choices were presented after each speech segment to assess story comprehension and to ensure attention during the task (Guo et al. 2025; McHaney et al. 2021; Reetzke et al. 2021). Feedback was presented in the format of “You got X correct out of 2 in this trial” (Figure 2C). Stimulus presentation was controlled by the E‐Prime 3.0 software (Schneider et al. 2002). Comprehension question performance for these participants was previously reported in Guo et al. (2025) and can be viewed in Supporting Information 1.

EEG responses were amplified and digitized with the BrainVision actiCHAMP amplifier and collected using BrainVision PyCorder 1.0.7 (Brain Products, Gilching, Germany) with 64‐channel actiCAP active electrodes (Brain Products) secured in an elastic cap (EasyCap; http://www.easycap.de/). Electrodes were placed on the scalp according to the International 10‐20 system, and a common ground was placed at the AFz electrode site. Electrode impedance was less than 25 kΩ for all channels. Responses were recorded at a sampling rate of 25 kHz.

### 2.4 Computation of the Dynamic F0 Waveform

The continuous speech F0 waveforms from the stimuli were extracted using empirical mode decomposition (Forte et al. 2017) for further analyses with the following steps. The stimuli were first downsampled to 8820 Hz, then low‐pass filtered at 1500 Hz and time‐shifted to account for filter delay. Silent periods between words, as defined by an envelope value < 10% of the maximal speech envelope, were identified and set to zero. The F0 of the stimuli was extracted using autocorrelation with a rectangular window of 50 ms and successive overlaps of 49 ms. Any frequencies detected outside the F0 range of 60–400 Hz, or any instantaneous frequencies with a variation larger than 10 Hz within 1 ms, were set to zero. The downsampled and filtered signal was also processed using a Hilbert–Huang transform (Huang and Pan 2006) that was used to extract intrinsic mode functions (IMFs) with two properties: (1) The quantity of extreme and zero values were equal or differed by one and (2) the mean of the upper and lower envelopes disappeared. This resulted in a linear superposition of the IMFs. The Hilbert spectrum of each IMF was determined and yielded the mode's instantaneous frequency. The F0 obtained from the autocorrelation of each speech segment was then compared with the instantaneous frequencies from the IMFs at each time point. All IMFs with an instantaneous frequency that differed by < 20% of the segment's autocorrelation‐F0 were identified, and the IMF with the greatest amplitude was selected as the F0 wavemode of that segment and time point (Huang and Pan 2006). The F0 waveform of the speech signal (Figure 2D) was thus derived by combining each timepoint through cosine crossfading functions with a 10‐ms window. The obtained F0 waveform was further downsampled to match the sampling rate of the EEG.

### 2.5 Estimation of Neural Coding of Dynamic F0

The raw EEG data were bandpass filtered between 70 and 1500 Hz using a hamming window with a filter order of 50. The data were then referenced to the average of channels at both mastoids. Next, the data were epoched from −5 to 70 s relative to the story segment onset, then down sampled to 1000 Hz for computational efficiency.

Neural coding of the F0 waveform was estimated separately for each subject using mTRF (Crosse et al. 2016) in MATLAB v2022a (Mathworks Inc.). We estimated the forward mapping of the continuous speech F0 waveform onto the EEG (Gnanateja et al. 2025; McHaney et al. 2021). Multivariate linear regressions were used to derive the forward temporal response function (TRF) between the F0 waveform and the EEG data in every channel at different time lags ranging from −25 to 50 ms. The TRFs were estimated using a 15‐fold cross‐validation, where 14 trials were used for TRF estimation in every iteration, which were averaged across trials and used to predict the neural response in the remaining trial. The Pearson's correlation coefficient between the predicted EEG and the observed EEG served as a metric of neural coding (*r‐*values). These steps were iterated 15 times to estimate the *r*‐values for all trials at each electrode. The *r*‐values were then averaged across all trials and served as the metric of similarity between the neural response and the F0‐predicted neural waveform in the continuous speech.

As high‐gamma power from intracranial EEG has been shown to correlate with the amplitude envelope of speech (Kubanek et al. 2013; Pasley et al. 2012), we conducted additional TRF analyses including envelope as a predictor to assess whether this feature may explain unique variance in our scalp‐EEG signals filtered between 70 and 1500 Hz and potentially bias the results. This analysis suggested no significant contribution from the envelope, and we therefore report the findings from the TRF model including only the dynamic F0 waveform (see Supporting Information 2 for details).

### 2.6 Pitch Accent Classification

The raw EEGs were downsampled to 128 Hz for computational efficiency, bandpass filtered from 1 to 15 Hz using minimum‐phase causal windowed sinc FIR filters, and then rereferenced to the average of two mastoid electrodes (Di Liberto et al. 2015, 2018; O'Sullivan et al. 2014). After rereferencing, electrical activity outside the ± 3 standard deviation range of the surrounding channels was rejected and 3D‐spline interpolated, and any large artefacts were suppressed using artefact subspace reconstruction (Dial et al. 2021; McHaney et al. 2021; Mullen et al. 2015). The artefact subspace reconstruction‐cleaned data were epoched from −5 to 70 s relative to the story segment onset. Independent component analysis was performed on the epoched data to identify and remove ocular and muscular artefacts and reconstruct the EEGs.

We then examined the extent to which the listeners' neural signatures reflected discrete pitch accent categories. To this end, we first aligned the EEGs with the timestamps of pitch accents in the audiobook labelled by a trained phonetician using the ToBI system (Silverman et al. 1992). Following the procedures in Llanos et al. (2021), we focused on the four most frequent pitch accents in American English (H*, L*, L + H*, and L* + H, with 1610, 318, 1188, and 322 tokens, respectively) and adopted an “accent‐related potential (ARP)” approach. That is, we extracted EEG epochs time‐locked to each pitch accent (from −0.5 to 0.5 s relative to the onset of each labelled token) and averaged all the epochs to each pitch accent, called the ARP. There were four ARPs for the four pitch accents for each participant.

Next, we classified the multidimensional ARP time series using EEGNet (Lawhern et al. 2018). EEGNet is a compact convolutional neural network that has been reported to learn useful features for classifying EEGs across different brain‐computer interface paradigms while allowing interpretation of the learned features. Compared with approaches such as computing separability or Euclidean distance scores of neural responses at individual channels (e.g., Llanos et al. 2021), a supervised machine‐learning classifier such as EEGNet is more robust to noise and may better capture nonlinear spatial and temporal dependencies that are critical for distinguishing pitch accent categories. Here, we used the EEGNet‐8,2 model (with eight temporal filters and two spatial filters per temporal filter) with a 0.5 dropout rate and trained it with 30‐fold cross‐validation on the ARP datasets for each age group separately. The ARP datasets consisted of 80 (4 pitch accents × 20 participants) and 76 (4 pitch accents × 19 participants) ARPs for younger and middle‐aged adults, respectively, with an equal number of ARPs for each pitch accent. Note that the model was not trained on the single‐trial EEGs for each pitch accent instance.

The cross‐validation proceeded as follows: For each age group, the entire ARP dataset was first randomly partitioned into 30 folds, each containing ~3.3% of all ARPs. We then trained the model using a leave‐one‐fold‐out procedure. First, we held out one fold as the test set. Then, the data from the remaining 29 folds were pooled together, and a random 15% of folds were further set aside to serve as the validation set. This validation set was used to monitor training and determine when the model achieved the best generalization performance without overfitting. All remaining ARPs were used to train the EEGNet model for a maximum of 300 epochs, using the cross‐entropy loss criterion, an initial learning rate of 0.0001, and a batch size of four. The learning rate decreased by a factor of 0.7 every 100 epochs, and 15 (~25%) of the channels were randomly zeroed out at each batch to curtail overfitting. At the end of each epoch, the model's cross‐entropy loss on the validation set was calculated, and the model from the epoch that achieved the lowest validation loss was selected to predict the hold‐out test set. This process iterated until all folds served as the test set.

After all iterations, every unique ARP (e.g., participant 1's ARP to L + H*) was predicted once by a model that had not seen it during the training period. However, with a single cross‐validation run, unrepresentative findings may arise due to a specific dataset split or any randomness during the training process. To minimize such a possibility, the whole 30‐fold cross‐validation procedure was further repeated 30 times, such that each unique ARP had 30 prediction outcomes. The ARP classification approach is depicted in Figure 2E.

### 2.7 Statistical Analyses

#### 2.7.1 Neural Phase Locking Analyses

We computed phase locking values from the FFR data to syllables to examine the ability for auditory nerve fibers to fire action potentials at the rate of F0 (Lachaux et al. 1999). Phase locking values were computed at every frequency from 30 to 3000 Hz (Ruggles et al. 2012) using a multitaper method. Equal draws from positive and negative polarities were used to calculate phase locking of the envelope FFR, with higher values indicating stronger phase locking. Phase locking values at F0 (100 Hz) were examined to confirm that phase locking values were significantly above the noise floor. The noise floor was defined as the average phase locking value from 85 to 95 Hz and 105 to 116 Hz. For each group, paired samples *t‐*tests were run to compare the phase locking value at F0 against the noise floor to determine that phase locking was reliably measured. Mann–Whitney *U* tests were used to examine the difference in phase locking values at F0 between age groups using the *rstatix* package (Kassambara 2021) in R version 4.3.1 (R Core Team 2023) due to the non‐normal distribution of the data, as confirmed by the Shapiro‐Wilks test (*p* < 0.05) and visual inspection of the quantile–quantile plot.

#### 2.7.2 Neural Coding of F0 in Continuous Speech

We performed a mass‐univariate, single‐sample *t*‐test on the *r*‐values, which reflect the similarity of the predicted EEG to the actual EEG as estimated by the TRFs, to test whether neural coding of dynamic F0 from the EEG to continuous speech was statistically greater than zero. The mass‐univariate *t*‐test is a cluster‐based permutation test implemented in the *Eelbrain* package (Brodbeck et al. 2023) that uses a *t*‐value equivalent to uncorrected *p* ≤ 0.05 as the cluster forming threshold. Clusters were based on the identification of meaningful effects across groups of adjacent electrodes that showed the same effect (Maris and Oostenveld 2007). A corrected *p*‐value was then computed for each cluster based on the cluster‐mass statistic in a null distribution from 10,000 random permutations (Maris and Oostenveld 2007). The largest *t*‐value from the cluster (*t*_max_) is reported as an estimate of effect size (Brodbeck, Hong, and Simon 2018).

We used the average *r‐*values across all electrodes for further statistical analysis. To understand the effects of middle age on neural coding of F0 in continuous speech, we analyzed the mean neural coding *r*‐value using a Welch's two‐sample *t*‐test, based on the normal distribution of the data (Shapiro‐Wilk test, *p* > 0.05) and non‐equal sample sizes between groups. The *t*‐test was calculated using the *rstatix* package (Kassambara 2021) in R version 4.3.1 (R Core Team 2023).

We also examined the effect of middle age on the TRFs averaged across all electrodes for neural coding of F0 in continuous speech. We applied a Hilbert transform on the TRFs to control for the periodic nature and response energy in the TRF because of the high degree of autocorrelation in the dynamic F0 waveform in order to focus on amplitude variations in the TRF rather than phase (Etard et al. 2019; Van Canneyt et al. 2021). The resultant TRFs were compared between age groups using a cluster‐based permutation test in the *Eelbrain* package (Brodbeck et al. 2023) using default parameters except for the analysis time window, which was modified to 0 to 40 ms.

#### 2.7.3 Pitch Accent Classification

We examined the classification accuracy and uncertainty of the EEGNet model. For a given input ARP, the model output probabilities that the ARP belonged to each of the four pitch accent classes. The ARP was correctly classified when its actual pitch accent class matched the class with the highest predicted probability. As the cross‐validation training was repeated 30 times, and hence, each unique ARP had 30 prediction outcomes, we calculated a single proportion of correct predictions across the 30 repetitions for each unique ARP. Also, we computed Shannon's entropy (Shannon 1948) using the output probabilities from the EEGNet model and averaged entropy values across the 30 repetitions for each unique ARP. A higher entropy indicated less confidence. The distribution of the model accuracy and entropy data was examined via Shapiro‐Wilk tests and quantile–quantile plots to determine the appropriate parametric or nonparametric test to compare group differences. A Welch's two‐sample *t*‐test was used to examine model accuracy as the data were considered to be normally distributed (*p* > 0.05). Model entropy data were not normally distributed (*p* = 0.01), which warranted the use of a Mann–Whitney *U* test to examine group differences. Both statistical tests were calculated using the *rstatix* package (Kassambara 2021) in R version 4.3.1 (R Core Team 2023).

To compare EEG channels relevant to ARP classification between younger and middle‐aged adults, we used the DeepLIFT algorithm (Shrikumar et al. 2017) with the Rescale rule as in (Lawhern et al. 2018) to interpret the features learned by EEGNet. For each ARP in the hold‐out set, DeepLIFT computed a 61 (channels) × 128 (timepoints) matrix of contribution scores denoting the amount of (positive or negative) evidence for the target pitch accent at each channel over time. As nonzero scores suggest relevance to classification, we first took their absolute values. We then averaged the score matrices of each unique ARP over the 30 repetitions and *z*‐transformed the scores within each matrix. Finally, for each participant, the matrices were averaged across the four pitch accents to derive a single 61 × 128 matrix summarizing the relative relevance of the channels to ARP classification over time. We focused on 0.0 to 0.5 s relative to ARP onset and performed mass‐univariate independent samples *t*‐tests to test if middle‐aged and younger adults differed in the average relevance in this time frame at individual electrodes, with *p*‐values Bonferroni‐corrected for multiple comparisons.

#### 2.7.4 Correlations

We calculated correlations to examine the relationship between phase locking to F0 and TRF amplitudes. TRF amplitude data were normally distributed (*p* = 0.267). Phase locking values showed a distribution with a positive skew (*p* < 0.001), warranting a nonparametric Spearman's rank correlation (*ρ*). We also calculated two additional correlations to examine the relationship between pitch accent classification entropy with phase locking to F0 and TRF amplitudes in a time period identified as significantly different between age groups through the permutation cluster analysis. Entropy was negatively skewed (*p* = 0.016), warranting Spearman's rank correlations. Significance values for correlations with entropy were adjusted using the Bonferroni method to correct for two comparisons. All correlations were estimated using the *rstatix* (Kassambara 2021) package in R version 4.3.1 (R Core Team 2023).

## 3 Results

### 3.1 Neural Coding of F0 Is Reduced in Middle Age

**FIGURE 3:**
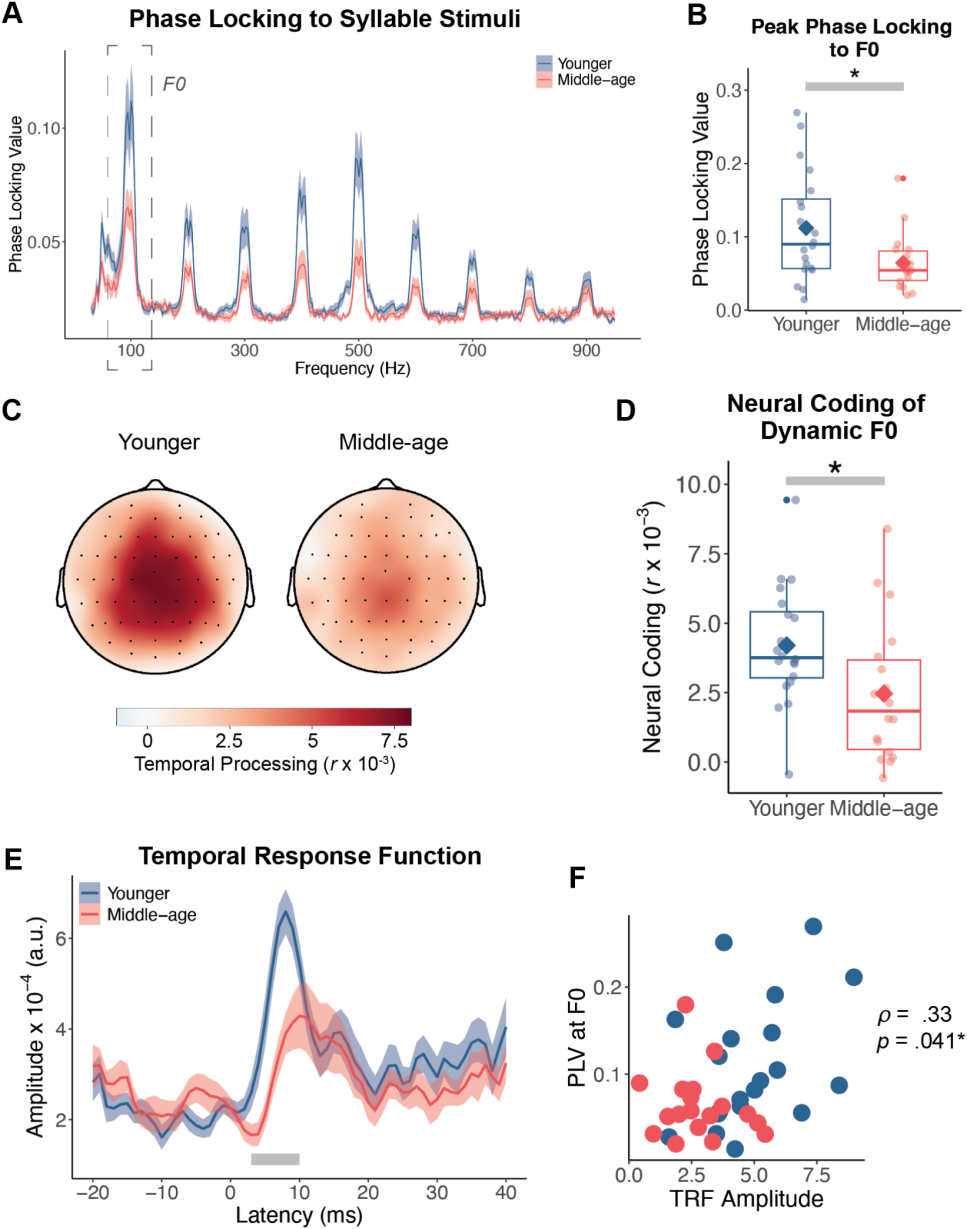
Neural coding of static and dynamic F0. (A) Phase locking values to static F0 and harmonics up to 900 Hz in syllable stimuli averaged across the three syllables. Solid lines represent average phase locking value in each group across frequencies and shaded region depicts standard error of the mean. (B) Phase locking values at F0 (100 Hz) were significantly lower in middle‐aged adults. Points represent individual participant phase locking values. The diamond reflects the mean in each group. (C) Neural coding of dynamic F0 in continuous speech was significantly lower in middle‐aged adults in a cluster of 60 electrodes. (D) Neural coding of F0 in continuous speech was significantly lower in middle‐aged adults. Points represent individual participant neural coding values (Pearson's *r*). The diamond reflects the mean in each group. (E) Hilbert envelope of the TRFs to dynamic F0 in continuous speech. TRFs were significantly different in a region spanning 3–9 ms, denoted by the solid grey bar. Solid lines represent the average TRF in each group and shaded region depicts standard error of the mean. (F) The average TRF amplitude across all electrodes in the significant region in panel E was significantly correlated with peak phase locking values (PLV) at F0 in panel B, showing that greater phase locking to static F0 was associated with larger TRF amplitudes to dynamic F0 in continuous speech.

We examined the extent to which younger and middle‐aged adults differed in neural coding of F0, to both syllable‐level stimuli with static F0 and continuous speech with dynamic F0. We first analyzed phase locking to the F0 (Figure 3A) derived from FFRs elicited to syllable stimuli with a static F0. Phase locking values at F0 were significantly above the noise floor in younger adults (*t*[19] = 5.450, *p* < 0.001, *d* = −1.219, 95% CI [−0.065, −0.021]) and middle‐aged adults (*t*[17] = 3.487, *p* = 0.003, *d* = −0.822, 95% CI [−0.032, −0.008]). Thus, phase locking at F0 was reliably measured in both age groups. We were particularly interested in the extent to which phase locking to F0 was reduced in middle‐age, and we found that phase locking at F0 was significantly lower in middle‐aged adults (*Mdn* = 0.054) relative to younger adults (*Mdn* = 0.090; *z* = 2.310, *p* = 0.021, *r* = 0.375; Figure 3B). These results indicate that the ability of neural ensembles to phase lock to the periodicity in the syllable stimuli was reduced in middle‐age.

Next, we compared neural coding of the dynamic F0 waveform in continuous speech stimuli across younger and middle‐aged adults. A mass‐univariate single‐sample *t*‐test identified 60 significant electrodes (*t*_max_ = 11.215, *p* < 0.001) based on meaningful effects across groups of adjacent electrodes demonstrating the same effect, indicating that neural coding of dynamic F0 was statistically greater than zero across age groups (Figure 3C). However, neural coding of dynamic F0 was significantly lower in middle‐aged adults (*M* = 0.002, *SD* = 0.003), relative to younger adults (*M* = 0.004, *SD* = 0.002; Welch's *t*(33.412) = 2.291, *p* = 0.028, *d* = 0.784, 95% CI [0.0002, 0.00033]; Figure 3D). These findings suggest that middle‐aged adults had reduced neural coding of F0 in continuous speech compared with younger adults, in addition to reduced F0 encoding in the syllable stimuli with static F0.

Younger adults had larger TRF amplitudes than middle‐aged adults for lags between 3 and 9 ms (*p* = 0.005; Figure 3E), which corresponded to responses that are primarily subcortical in nature (Hashimoto et al. 1981; King et al. 2016; Skoe and Kraus 2010). We then averaged the TRF amplitudes across the 3–9 ms region for each participant and ran a correlation with phase locking values to static F0 in the syllable stimuli. This revealed a significant positive correlation between mean TRF amplitudes and phase locking values to static F0 (*ρ* = 0.33, *p* = 0.041; Figure 3F). Taken together, these results demonstrate that middle‐aged adults have reduced neural coding of pitch cues, at both the syllable level and to more naturalistic speech stimuli, and that individuals with poorer neural coding of static F0 were more likely to have poorer neural coding of dynamic F0.

### 3.2 Pitch Accent Classification in Middle Age Is as Accurate but Less Certain Than Younger Adults

**FIGURE 4:**
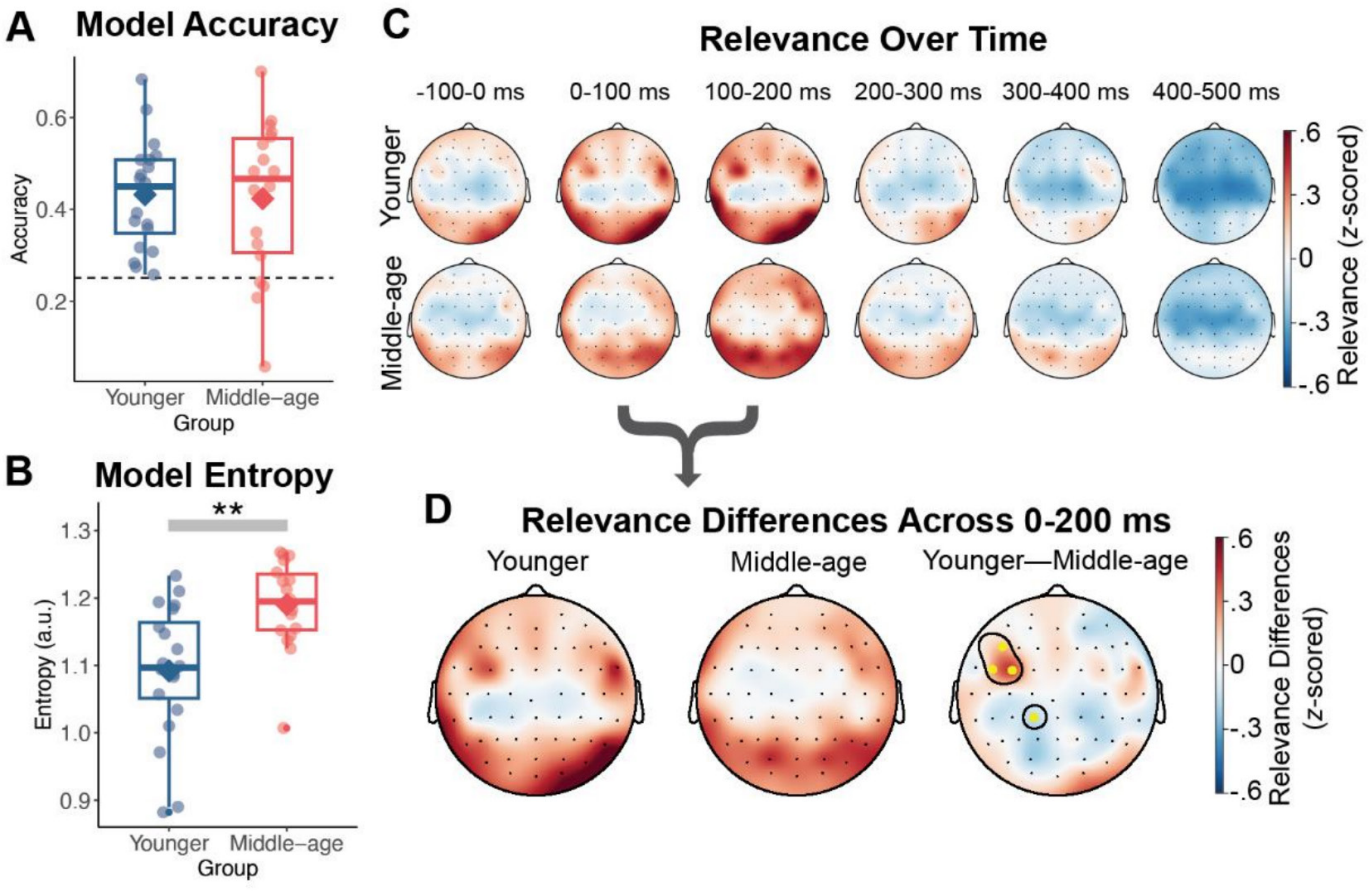
Pitch accent classification. (A–B) Model classification accuracy did not differ between younger and middle‐aged adults, but model entropy was significantly higher in middle age. Individual participant values are denoted by points. Chance level accuracy is denoted by the horizontal dashed line. (C) Electrode relevance over time in 100 ms blocks, time‐locked to pitch accent onset. Electrodes were most relevant in the 0–200‐ms time period for both groups. Darker red colours indicate more relevance for pitch accent classification. (D) In the 0–200‐ms time period, younger adults showed more relevance for classification in a cluster of frontocentral electrodes, while middle‐aged adults showed a significant central‐parietal cluster for classification. Significant clusters are highlighted in yellow and outlined by a dark line.

We examined the extent to which EEG responses to continuous speech reflected the four most frequent pitch‐accent categories in the audiobook using an ARP approach. ARP classification accuracy from the EEGNet (Lawhern et al. 2018) was not different between younger (*M* = 0.432, *SD* = 0.116) and middle‐aged adults (*M* = 0.424, *SD* = 0.168; *t*[29.852] = 0.181, *p* = 0.858, *d* = 0.059, 95% CI [−0.088, 0.105]; Figure 4A). However, the model entropy, which can be interpreted as classification uncertainty, was significantly higher in middle‐aged (*Mdn* = 1.195) relative to younger adults (*Mdn* = 1.096; *z* = −3.274, *p* = 0.001, *r* = 0.531; Figure 4B). These findings suggest that while classifiers trained on ARPs in middle‐aged adults were able to correctly identify the pitch accent similar to younger adults, the middle‐aged adult model had significantly more difficulty discriminating neural responses between pitch accents, yielding larger entropy values compared with younger adults.

We then analysed the EEG features learned by the EEGNet model (Lawhern et al. 2018) using the DeepLIFT algorithm (Shrikumar et al. 2017) to quantify which spatio‐temporal features of the ARP were relevant to pitch accent classification and whether the relevance patterns differed between younger and middle‐aged adults. Topographic maps of the average relevance in 100‐ms intervals over the duration of the ARP are depicted in Figure 4C for each electrode. For both age groups, the overall relevance value showed a similar trajectory over time: higher relevance in the first 200 ms post onset followed by a decrease through 500 ms. This pattern was consistent with the previous findings that ARPs to different pitch accents were most separable within the first 200 ms of ARP onset (Llanos et al. 2021). Here, younger adults also appeared to have more relevant electrodes in the frontotemporal areas in the time windows of 0–100 and 100–200 ms post pitch accent prominence. Therefore, we focused the remainder of our analyses on the electrode relevance across 0–200 ms.

To test the effect of age group differences in electrode relevance across 0–200 ms, we ran a repeated‐measures ANOVA with age group as a between‐subjects factor and electrode as a within‐subjects factor. The results revealed a significant interaction of age group and electrode (*F*[60,2317] = 3.02, *p* < 0.001) and a significant main effect of electrode (*F*[60,2317] = 14.05, *p* < 0.001), but no main effect of age group (*F*[1,2317] = 0.32, *p* = 0.573). We then ran a mass‐univariate independent samples *t*‐test on the 0–200‐ms topographies, controlling for multiple comparisons, to further examine the group differences across electrodes (Figure 4D). A prominent difference between younger and middle‐aged adults was that younger adults showed stronger relevance in a cluster of frontocentral electrodes (*p*s < 0.05), while middle‐aged adults showed stronger relevance for a central‐parietal cluster (*p*s < 0.05). These findings indicate that different regions of electrodes were relevant for pitch accent classification, depending on age group. Thus, these differing regions relevant for classification may underlie some of the differences observed in model entropy for classifying pitch accents between younger and middle‐aged adults.

### 3.3 Greater Phase Locking to F0 Relates to Lower Model Entropy

**FIGURE 5:**
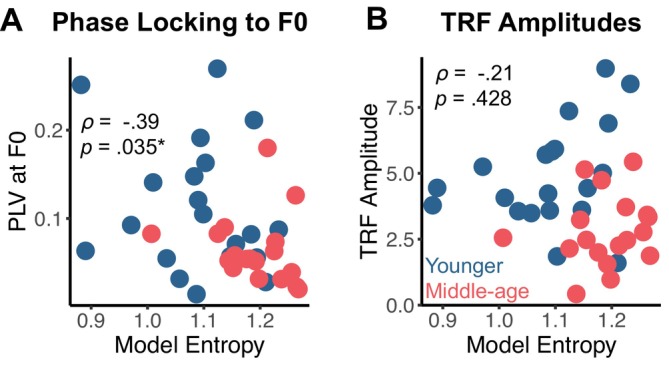
Relationships between neural coding of F0 and pitch accent classification entropy. (A) Model entropy was correlated with peak phase locking values at F0 (Spearman's *ρ* = −0.39, *p*‐adjusted = 0.035). (B) Model entropy was not correlated with TRF amplitudes. Points denote individual participants.

We explored the extent to which model entropy may be explained by neural coding of F0. We examined Spearman's rank correlations of entropy with phase locking to F0 in syllables and TRF amplitudes to F0 in continuous speech (Figure 5). Entropy was significantly correlated with phase locking to F0 (*ρ* = −0.39, Bonferroni‐adjusted *p* = 0.035) but not TRF amplitudes (*ρ* = −0.21, Bonferroni‐adjusted *p* = 0.428). The correlation between entropy and phase locking to F0 suggests that individuals who had poorer phase locking to F0 were more likely to have greater model entropy or more uncertain pitch accent classification. This evidence suggests that neural coding of F0 may impact neural processing of pitch accents.

## 4 Discussion

Middle‐age is ripe with increasing complaints of speech perception challenges, even when there are no overt signs of hearing loss (Demeester et al. 2012). The reasons for this are unclear, but mounting evidence suggests that reduced neural coding of F0 may contribute to speech perception difficulties with advancing age (Anderson et al. 2011; Ruggles et al. 2012; Van Canneyt et al. 2021). Neural coding of F0 has commonly been measured to repeated instances of isolated syllables with a static, non‐changing F0 (e.g., Anderson et al. 2011; Ruggles et al. 2012). However, isolated syllables with a static F0 are a poor representation of naturalistic speech contexts. Recently, studies have transitioned to examining neural coding of F0 in continuous speech stimuli (Forte et al. 2017) and have demonstrated a decline in F0 coding with advancing age (Van Canneyt et al. 2021). Here, we examined neural coding of F0 in discrete syllables with a static F0 and to continuous speech stimuli with a more naturalistic, dynamic F0 within the same individuals to examine the extent to which neural coding of F0 is reduced in middle‐age and whether these measures capture similar pitch processing mechanisms. Additionally, we leveraged the wealth of information available in continuous speech stimuli to examine neural processing of abstract pitch accents that rely on changes in F0 contours within words and syllables to understand the downstream effects of neural coding of F0 on higher‐order prosodic features in middle‐age.

Consistent with prior research, we observed declines in neural coding of F0 in middle‐aged adults to both static F0 in isolated syllables (Ruggles et al. 2012) and to naturalistic, continuous speech with dynamic F0 (Van Canneyt et al. 2021) compared with younger adults. Importantly, our results confirm that middle‐aged adults show reduced phase locking to F0, which was previously observed by Ruggles et al. (2012) in a smaller sample of middle‐aged adults using traditional isolated syllables. These findings validate that middle age is accompanied by changes in neural coding of pitch cues, such that neuronal ensembles are less able to spontaneously fire at the F0 of speech. Moreover, we demonstrated a relationship between neural coding measures to both stimulus types. That is, individuals with greater phase locking to F0 in syllables were more likely to have larger TRF amplitudes to F0 in continuous speech, suggesting that these two measures reflect shared pitch processing mechanisms. However, it is worth noting that the correlation was not very high, which suggests that syllabic and continuous speech stimuli target slightly different mechanisms, possibly due to differences in the range of F0.

One possibility is that phase locking to F0 in syllables and neural coding of F0 in continuous speech could be differentially affected by attention. Phase locking to F0 in syllables was measured in a passive manner using FFR, while EEG to F0 in continuous speech was collected while participants were actively engaged in a task (i.e., periodically prompted to answer comprehension questions about the speech stimuli). There has been some research to indicate that attention may influence the FFR to syllables (Hartmann and Weisz 2019; Holmes et al. 2018; Hoormann et al. 2004; Lehmann and Schönwiesner 2014), while some evidence suggests no effect of attention (Varghese et al. 2015). Studies using a selective attention task with two auditory stimuli with continuous speech have shown attentional modulation of F0 encoding (Etard et al. 2019; Forte et al. 2017; Riegel et al. 2024; Schüller et al. 2023), while a study using a single auditory stimulus while switching attention between auditory and visual stimuli did not show attention effects (Xie 2025). It is possible that such attention effects in the neural coding of F0 in continuous speech might primarily be dependent on competition between two auditory stimuli. Thus, the slightly different patterns of results in the two tasks in the current study might not be mediated by attentional differences, but rather from the wide range of F0 variation in continuous speech compared with relatively static F0 in syllables.

Emerging evidence implicates both subcortical and cortical contributions to the FFR (Coffey et al. 2016, 2019; Gnanateja et al. 2021; Kulasingham et al. 2020; Schüller et al. 2024), whereas the FFR was historically considered to have only subcortical generators (Moushegian et al. 1973; Sohmer et al. 1977). An argument could be made that attention might have mediated only the cortical components of the FFR. We did not explicitly disentangle cortical responses from the FFR in the current study. It is possible that FFRs to continuous speech, especially for low F0 sections, could have auditory cortical contributions (Gnanateja et al. 2025; Schüller et al. 2023, 2024), which may have been partially modulated by attention. However, we cannot completely rule out the role of cortical responses in our FFRs to continuous speech, and thus top‐down effects of attention on the neural coding of F0 in continuous speech. Further research is necessary to disentangle the interactions of attention, age, and neural coding of F0.

The age‐related changes in pitch encoding that we observed were likely not a direct result of reduced hearing sensitivity with age, as we matched hearing thresholds between younger and middle‐aged adults. Therefore, the lower F0 encoding in middle‐aged adults could be a result of age‐related changes in temporal envelope processing (Grose et al. 2009; He et al. 2008; Helfer and Vargo 2009) and fine‐structure processing (Grose and Mamo 2010; Lorenzi et al. 2006; Moore et al. 2012). This affected temporal envelope and fine‐structure processing might be mediated by reduced synaptic efficiency and phase‐locking that might be caused by age‐related declines or life‐long noise exposure (Kujawa and Liberman 2009; Schmiedt et al. 1996), which do not necessarily manifest as elevated hearing thresholds on the standard audiogram. Thus, reduced phase locking to pitch cues might be one of the important factors driving suprathreshold processing difficulties reported by middle‐aged adults (Helfer and Jesse 2021), such as difficulty segregating target speech in multi‐talker listening situations, resulting in increased listening effort.

Beyond pitch cues being used to segregate talkers in multi‐talker listening scenarios, they are also used to convey intent through pitch accents. We leveraged the continuous speech stimuli to examine neural processing of pitch accents and their possible connection to neural coding of F0. Not only did middle‐aged adults show reduced neural coding of F0 compared with younger adults but they also showed more uncertain neural responses to pitch accents. Classifiers trained on ARPs in middle‐aged adults accurately identified the correct pitch accent, similar to the one trained on younger adults. However, the middle‐aged adult model had more difficulty discriminating between pitch accents, resulting in higher entropy than in younger adults. It is important to note that while these findings of similar accuracy yet higher entropy in middle age may seem contradictory, these metrics capture two distinct aspects of neural processing. For each ARP, the model estimated the probability that the ARP was each of the four pitch accents. The pitch accent with the highest probability was then selected as the most likely pitch accent category. Accuracy only considers the pitch accent class with the highest probability value, while entropy considers the probabilities of all four pitch accent classes to quantify an overall uncertainty in its prediction. Our entropy results indicated that the probability distributions across the four pitch accent categories were more similar within middle‐aged adults, even though the model ultimately made the correct choice as often as it did for younger adults.

Our finding that probability distributions were more similar across pitch accent classes within middle‐aged adults may support the age‐related neural dedifferentiation hypothesis (Koen and Rugg 2019; Park et al. 2004). This hypothesis suggests that pitch accent processing may show greater uncertainty in middle‐age due to less distinctive and more correlated neural activity across the cortex. Age‐related neural dedifferentiation has traditionally been examined in the context of older adults, but a separate recent study also suggests that this may occur even in middle‐aged adults during speech perception (Guo et al. 2025). Our findings suggest that middle‐aged adults might have more variable neural responses to pitch accents, providing further evidence for neural dedifferentiation in middle‐age. Future studies could leverage neuroimaging approaches to better identify neural changes in processing pitch accents associate with age that may provide additional support for neural dedifferentiation of pitch accents, and more broadly, speech processing in middle‐age.

Neural coding of static F0 in syllables (i.e., phase locking) was related to pitch accent model entropy, regardless of age group. This negative relationship suggests that individuals with poorer phase locking to static F0 in syllables were more likely to show greater uncertainty in processing pitch accents. In middle‐aged adults, this may reflect early manifestations of neural dedifferentiation, where declining phase locking precision leads to less distinct neural representations of prosodic features, such as pitch accents. Further, reduced phase locking to F0 may drive compensatory recruitment of additional neural resources to process pitch accents due to less specialized processing in Heschl's Gyrus, potentially contributing to the observed higher entropy. Interestingly, TRF amplitudes from EEG responses to dynamic F0 in continuous speech were not directly related to pitch accent model entropy, even though these measures were estimated from the same continuous speech stimuli, but albeit different features of the stimuli. The significant relationship for phase locking values but not TRF amplitudes may reflect the underlying metric itself. Phase locking of F0 reflects the ability for auditory nerve fibers to precisely fire action potentials at the rate of F0 (Lachaux et al. 1999). In contrast, the TRF reflects an impulse response of the auditory system at F0 (Crosse et al. 2016; Ding and Simon 2012), which may serve as a broader measure of the overall sensitivity of the auditory system to dynamic F0 amplitude and phase in continuous speech. Model entropy may have demonstrated a direct relationship with phase locking as it is a more focalized metric of pitch cue encoding. Taken together, our findings indicate that reduced neural coding of F0 in middle age may result in more variable neural processing of pitch accents.

The relationships between F0 coding measures and pitch accent processing give us important insights into altered speech feature processing in middle‐aged adults. Pitch accents are primarily processed at the cortical level in Heschl's gyrus and are higher order linguistic features that rely on precise encoding of the F0 information in speech (Gnanateja et al. 2025). However, we did not see a very strong one‐to‐one relationship between F0 encoding in continuous speech and pitch accent processing. The lack of a strong correlation between F0 encoding and pitch accent processing, coupled with lower F0 encoding and higher entropy yet similar accuracies for pitch accent processing in middle age, potentially suggests the presence of compensatory changes in the auditory cortex and higher structures. The auditory cortex, which is the primary site for pitch accent processing (Gnanateja et al. 2025), might be compensating for reduced phase‐locking information received from the lower auditory centers. While this potential compensation resulted in similar pitch accent classification in both younger and middle‐aged adults, the encoding of pitch accent representations was more variable and not as robust in middle age, as evidenced by the higher entropy.

Further, higher entropy for pitch accent classification in middle‐aged adults may reflect subtle differences in listening strategies. Middle‐aged adults may employ secondary auditory or extra‐auditory cortical regions when processing spoken language, leading to more variations in their neural responses to pitch accents, and hence, higher classification uncertainty as we observed in the present study. Prior research suggests that older adults recruit extra‐auditory regions for processing speech in noisy environments (Brodbeck, Presacco, et al. 2018; Du et al. 2016). Thus, middle‐aged adults may use a broader network of brain areas or alternate between different linguistic or attentional strategies, leading to more variable neural responses to pitch accents, which could suggest that extra‐auditory recruitment for listening may occur as early as middle age. Such compensatory recruitment might be linked to increases in listening effort that have been observed in the absence of overt hearing loss (Degeest et al. 2015; Helfer et al. 2020; McHaney et al. 2024; Zink et al. 2025). Future studies should leverage measures such as pupillometry or subjective listening ratings in concert with the measures in the current study to examine the extent to which middle‐aged adults exert more listening effort during pitch accent processing to better understand the extent to which variable neural responses to pitch accents, listening effort, and compensatory recruitment are related. Additionally, carefully designed studies that require participants to categorize pitch accents could use perceptual decision‐making models, such as drift‐diffusion models (Ratcliff et al. 2016; Ratcliff and McKoon 2008). Findings from such studies would help to inform the strategies listeners use to make decisions about pitch accent patterns in speech, paving the way to understanding whether middle‐aged adults employ different linguistic or attentional strategies that might contribute to observed variations in neural responses to pitch accents and changes in listening effort.

### 4.1 Conclusion

In conclusion, these results demonstrate that middle‐aged adults have reduced neural coding of F0, which may lead to downstream difficulties processing abstract linguistic pitch accents. Neural responses to pitch accents were less robust in middle‐aged adults. Diminished neural representations of pitch accents may require the listener to deploy additional cognitive resources for accurate speech processing and hence result in greater listening effort and perceived listening difficulty in middle‐aged adults with normal hearing. These findings align with other recent studies in middle age demonstrating greater listening effort prior to behavioral changes in speech perception (Zink et al. 2025) and neural dedifferentiation of phonemes during spoken language processing (Guo et al. 2025), further highlighting the need to consider the interplay of subcortical and cortical loci for speech processing challenges in middle‐aged adults without hearing loss.

## Author Contributions

**Jacie R. McHaney:** conceptualization, data curation, formal analysis, funding acquisition, investigation, methodology, project administration, validation, visualization, writing – original draft, writing – review and editing. **Zhe‐chen Guo:** data curation, formal analysis, methodology, validation, writing – original draft, writing – review and editing. **G. Nike Gnanateja:** formal analysis, methodology, software, validation, writing – review and editing. **Aravindakshan Parthasarathy:** funding acquisition, project administration, resources, supervision, writing – review and editing. **Bharath Chandrasekaran:** conceptualization, funding acquisition, project administration, resources, supervision, validation, writing – review and editing.

## Conflicts of Interest

The authors declare no conflicts of interest.

## Peer Review

The peer review history for this article is available at https://www.webofscience.com/api/gateway/wos/peer‐review/10.1111/ejn.70285.

## Supporting information


**Data S1:** Continuous speech comprehension scores.

**Data S2:** Temporal response function analysis including amplitude envelope.

## Data Availability

All data and code used to analyze data in this experiment are available on the Open Science Framework at https://doi.org/10.17605/OSF.IO/9RDFJ.
