# Supplementary material for: Neural Coding of Fundamental Frequency and Processing of Discrete Pitch Accents in Middle Age
Source: Eur J Neurosci. 2025 Oct 30;62(9):e70285. doi: 10.1111/ejn.70285 (PMC12573739; doi:10.1111/ejn.70285)
Supplement: Supplementary file 1 — Data S1: Continuous speech comprehension scores. [file EJN-62-0-s002.pdf]

Continuous Speech Comprehension Scores

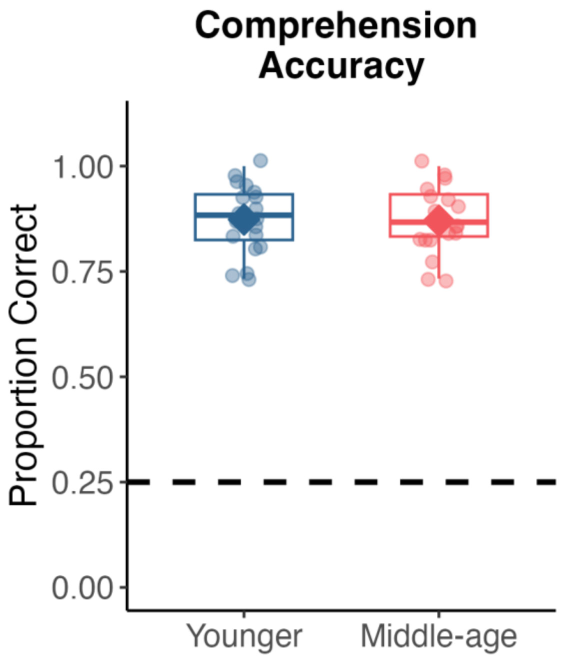

The above figure displays scores on the comprehension questions presented during the continuous speech EEG task. Age groups did not significantly differ in the proportion of correct responses on these questions ($t(35.868) = 0.116$, $p = .908$, $d = 0.038$, 95% CI[-0.050, 0.056]). Points represent individual participant comprehension scores. The diamond reflects the mean in each group. The horizontal dashed line represents chance accuracy (.25). A subset of these data were previously reported in Guo et al. (2025).
