# Supplementary material for: Neural Coding of Fundamental Frequency and Processing of Discrete Pitch Accents in Middle Age
Source: Eur J Neurosci. 2025 Oct 30;62(9):e70285. doi: 10.1111/ejn.70285 (PMC12573739; doi:10.1111/ejn.70285)
Supplement: Supplementary file 2 — Data S2: Temporal response function analysis including amplitude envelope. [file EJN-62-0-s001.pdf]

Temporal Response Function Analysis Including Amplitude Envelope

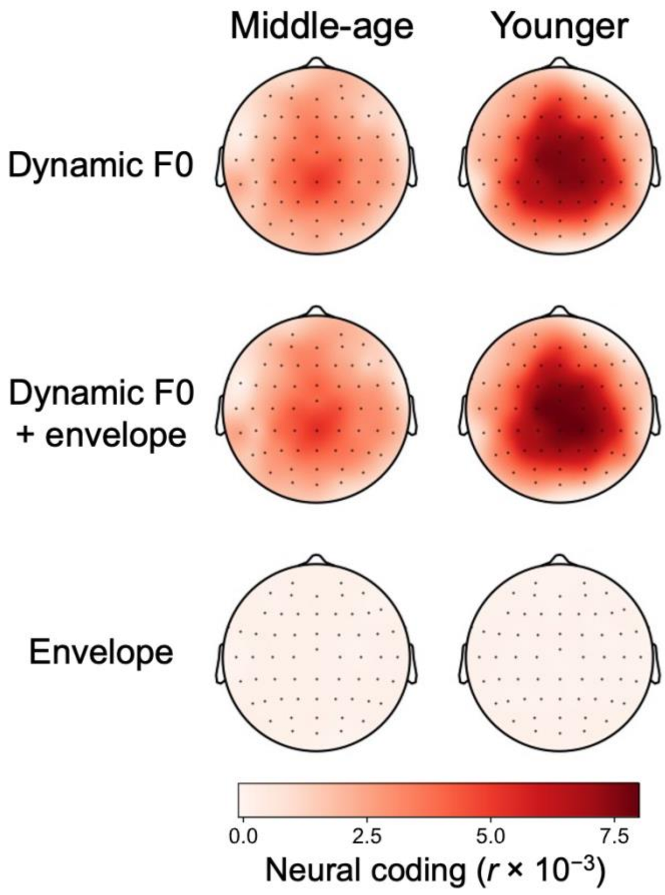

To evaluate whether the amplitude envelope may contribute to the prediction of EEG responses and bias the results, we re-estimated TRF models including the following two sets of predictors: 1) amplitude envelope only; 2) both dynamic F0 waveform and envelope. As shown in the figure above, the neural coding values of the envelope-only model were low and its average neural coding across electrodes ($M = 6.469 \times 10^{-5}$, $SD = 8.372 \times 10^{-5}$) was significantly lower than that of the combined model with both predictors ($M = 3.575 \times 10^{-3}$, $SD = 2.431 \times 10^{-3}$; $t(37) = -8.863$, $p < .001$, $d = -1.438$, 95% CI $[-4.313 \times 10^{-3}, -2.708 \times 10^{-3}]$). On the other hand, the model including only dynamic F0 ($M = 3.378 \times 10^{-3}$, $SD = 2.448 \times 10^{-3}$) did not differ significantly in average neural coding strength from the combined model ($t(37) = -.426$, $p = .673$, $d = -.069$, 95% CI $[-1.132 \times 10^{-3}, .739 \times 10^{-3}]$). These results suggested that the envelope feature did not add to explaining unique variance in the neural responses and hence was unlikely to confound the findings.
